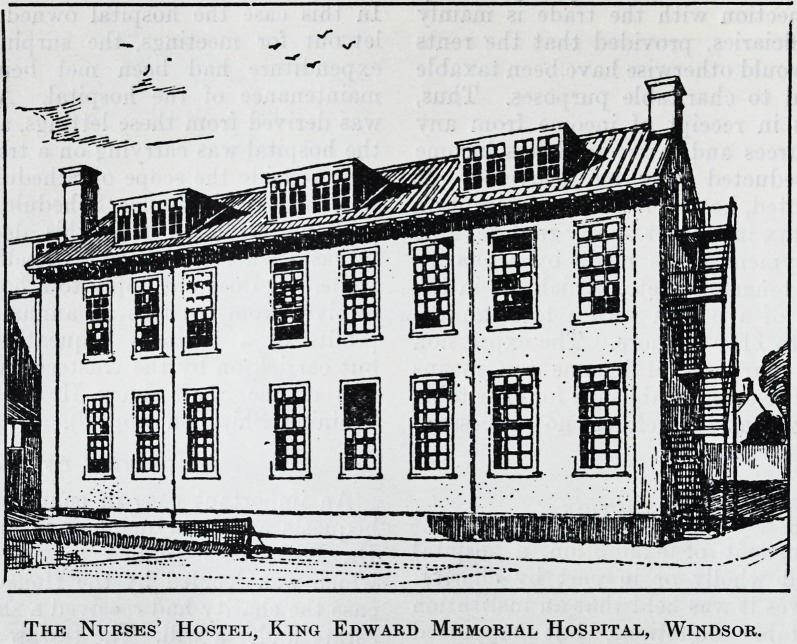# The Queen at the New Nurses' Hostel, Windsor

**Published:** 1924-05

**Authors:** 


### THE QUEEN AND THE NURSES' HOSTEL AT WINDSOR.

Driving from Windsor Castle in an open carriage, preceded by postilion and outrider, the Queen, on April 5, laid the foundation-stone of the New Nurses' Hostel of the King Edward VII. Memorial Hospital, Windsor, of which the King is Patron and the Prince of Wales is President. The new building, which is being erected by Messrs. Norris and Company, of Sunninghill, under the designs of Mr. H. Spink, the architect, is expected to be ready by August. The Hostel is to constitute the local War Memorial, jointly with a memorial to the late Sir William Shipley, a former chairman of the hospital. The erection of this building will enormously facilitate the work of the hospital in providing more space for the treatment of patients, as there is now permanently a long waiting list. It is the authorities' intention also to fill a long-felt want by providing for some twelve or fifteen paying patients of moderate means. The Hostel, which forms a new wing to the existing building, will liberate the rooms in the main block now occupied by the nursing staff and free them for the isolation department, and for X-ray and Insulin treatment. The building is being so constructed as to permit—when money is forthcoming—of being easily converted into additional hospital wards by the removal of partitions when a further extension is required and a fresh building shall have been put up to house the nurses. At present the hospital is sorely needing money.

The Nurses' Hostel, King Edward Memorial Hospital, Windsor.

Her Majesty was received by the Chairman of the Hospital, Mr. Richard Bentley, and others. Before laying the foundation-stone with a silver trowel Mr. Spink, the architect, and Mr. Yates, the builder, were presented to Her Majesty. After a prayer had been offered by the chaplain, the Queen signed the visitors' book, which contains many royal autographs. She then received a number of presents, the first being inscribed: "To Her Majesty from a Poor Woman." At the conclusion of the ceremony the Queen expressed a wish to see one of the housemaids of Windsor Castle, who was in the Hospital awaiting operation, and was conducted round the wards. After a tour of over an hour, during which she spoke to each of the eighty in-patients, the Queen left, expressing her deep interest and satisfaction at her visit and warmly complimenting the medical and nursing staffs.

### A Charity Information Bureau.

A Medical Charities Information Bureau has been formed at Newcastle-on-Tyne, where a fund is being raised for the Princess Mary Maternity and other Tyneside hospitals. The Maternity Hospital will be finished in June, with 95 beds, and still needs £5,000 to complete the £20,000. The £15,000 in hand has been raised during the past year, and includes a legacy of £50 from the late Matron. Representatives of each hospital will be on the committee of the Charity Information Bureau.

## Figures and Tables

**Figure f1:**